# Differentially Expressed Mitochondrial Proteins in Human MCF7 Breast Cancer Cells Resistant to Paclitaxel

**DOI:** 10.3390/ijms20122986

**Published:** 2019-06-19

**Authors:** Petr Daniel, Petr Halada, Michael Jelínek, Kamila Balušíková, Jan Kovář

**Affiliations:** 1 Department of Biochemistry, Cell and Molecular Biology, Third Faculty of Medicine, Charles University, Ruská 87, 100 00 Prague, Czech Republic; michael.j@email.cz (M.J.); kamila.balusikova@lf3.cuni.cz (K.B.); 2 Laboratory of Molecular Structure Characterization, Institute of Microbiology, v.v.i., Vídeňská 1083, 142 20 Prague, Czech Republic; halada@biomed.cas.cz

**Keywords:** breast cancer cells, paclitaxel resistance, mitochondria, two-dimensional electrophoresis, carbamoyl-phosphate synthetase 1 (CPS1), abhydrolase-domain containing protein 11 (ABHD11), cathepsin D, ATPase family AAA-domain containing protein 3A and 3B (ATAD3A, 3B)

## Abstract

Identification of novel proteins with changed expression in resistant cancer cells could be helpful in elucidation mechanisms involved in the development of acquired resistance to paclitaxel. In this study, we carried out a 2D-PAGE using the mitochondrial-enriched fraction from paclitaxel-resistant MCF7/PacR cells compared to original paclitaxel-sensitive MCF7 breast cancer cells. Differentially expressed proteins were identified employing mass spectrometry. We found that lysosomal cathepsin D and mitochondrial abhydrolase-domain containing protein 11 (ABHD11) had decreased expression in MCF7/PacR cells. On the other hand, mitochondrial carbamoyl-phosphate synthetase 1 (CPS1) and ATPase family AAA-domain containing protein 3A and 3B (ATAD3A, ATAD3B) were overexpressed in MCF7/PacR cells. Further, we showed that there was no difference in localization of CPS1 in MCF7 and MCF7/PacR cells. We demonstrated a significant increase in the number of CPS1 positive MCF7/PacR cells, using FACS analysis, compared to the number of CPS1 positive MCF7 cells. Silencing of CPS1 expression by specific siRNA had no significant effect on the resistance of MCF7/PacR cells to paclitaxel. To summarize, we identified several novel proteins of a mitochondrial fraction whose role in acquired resistance to paclitaxel in breast cancer cells should be further assessed.

## 1. Introduction

Breast cancer is the most commonly diagnosed cancer in women with 1.67 × 10^6^ newly diagnosed cases and nearly 5 × 10^5^ deaths per year [1]. Because of the heterogeneous nature of breast cancer, chemotherapy is based on various anticancer agents [2]. Specific chemotherapy by aromatase inhibitors (i.e., Tamoxifen ®) for estrogen-positive breast cancer or monoclonal antibody (i.e., Trastuzumab ®) for HER2 positive breast cancer can be useful when used in combination with anthracyclines (Doxorubicin, Epirubicin) and taxanes (Paclitaxel, Taxol®, and Docetaxel, Taxotere®) [3,4]. Taxanes and anthracyclines are the preferred choices for the treatment of triple-negative breast cancer [4,5].

The anticancer effect of paclitaxel (Taxol ®) was discovered while testing yew (*Taxus brevifolia*) bark extract on cancer cell proliferation [6]. It was shown that taxanes, i.e., taxol-related compounds, exert their effects through binding to the beta subunit of tubulin [7,8], and thus stabilizing microtubules [9]. Cells exposed to clinical doses of taxanes trigger the spindle assembly checkpoint and subsequently initiate apoptosis [10]. However, the cells arrested in mitosis may exit mitosis and die via various mechanisms, survive as senescent cells, or may be able to duplicate as aneuploid cells [11].

Acquired resistance has been extensively studied in cultured paclitaxel-resistant cells of breast [12,13,14,15], ovarian [16,17], lung [18], prostate [19,20] and hepatocellular [21] origins. The most common finding is that paclitaxel-resistant cells induce the expression of one or more members of the ATP-binding cassette (ABC) transporter family, particularly the ABCB1 transporter (P-glycoprotein) [14,22]. Other broadly discussed mechanisms that can contribute to taxane resistance are mutations or alterations in the expression of both tubulin subunits [21,23], expression of detoxifying enzymes [24], and expression of microtubule-associated proteins [25]. Other mechanisms of taxane resistance have also been discussed [26]. 

In order to elucidate the molecular mechanisms of acquired resistance to taxanes, we established an MCF7/PacR subline resistant to death-inducing concentrations of paclitaxel (300 nM) through multistep adaptation from the original paclitaxel-sensitive MCF7 cell line [14]. We analyzed the expression of 49 known human ABC transporters at the mRNA and protein level in paclitaxel-sensitive MCF7 cells versus paclitaxel-resistant MCF7/PacR cells [14]. ABCB1, ABCB4, ABCC2, and ABCC3 transporters were found to be overexpressed in MCF7/PacR cells. Concerning transporter ABCB1, it was shown to transport paclitaxel out of cancer cells [27]. Molecular docking using a humanized model of mouse ABCB1 transporter showed the binding of paclitaxel into its large binding cavity and transport of taxanes out of MCF7/PacR cells [28]. Recently, interaction and model of paclitaxel transport by ABCB1 transporter was elegantly presented [29]. Beside enhanced transporter activity, we reported that thyroid-hormone interacting protein 6 (TRIP6) is markedly upregulated (650%) whereas lysosomal protease cathepsin D and heat shock protein 27 (HSP27) are both downregulated (28% and 47%, respectively) in MCF/PacR cells [30].

Downregulation of ABCB1 expression leads to only a partial decrease in resistance to paclitaxel. It indicates that more mechanisms might be involved in paclitaxel resistance in MCF7/PacR cells [28,30]. Mitochondria, where up to 1,900 constituent proteins are present [31], play an essential role in apoptosis regulation [32] and thus it is reasonable to test the possible involvement of mitochondrial proteins in resistance to paclitaxel. Mitochondria are also essential for cell metabolism and respiration [33], cell signaling [34], and other cell functions [35,36]. Therefore, in the present study, we tested differences in protein expression in the mitochondrial fraction of paclitaxel-resistant MCF7/PacR compared to paclitaxel-sensitive MCF7 breast cancer cells, using two-dimensional polyacrylamide gel electrophoresis (2D-PAGE). We found that mitochondrial carbamoyl-phosphate synthetase I (CPS1) was significantly overexpressed (494%) in MCF7/PacR cells. It seems that its expression was not caused by increased expression in each MCF7/PacR cell but rather due to an increase in the number of CPS1 positive MCF7/PacR cells. Downregulation of CPS1 expression in MCF7/PacR cells had no effect on resistance to paclitaxel. Other proteins with changed expression were mitochondrial lipase abhydrolase domain-containing protein 11 (ABHD11) (downregulation to 68%) and ATPase family AAA-domain containing protein 3A and 3B (ATAD3A/3B) (downregulation to 154% was statistically nonsignificant). Probably as a product of contamination of mitochondrial fraction with lysosomes, we also detected lysosomal protease cathepsin D (CTSD) downregulated in MCF7/PacR cells (19%).

## 2. Results

### 2.1. Isolation of Mitochondrial Fraction

To isolate mitochondria from MCF7 breast cancer cells, we used a Mitochondria QProteome® Mitochondria Isolation Kit (Qiagen) (see Section 4). Obtained mitochondrial fractions were termed “high-purity mitochondrial fraction”. 

**Figure 1:**
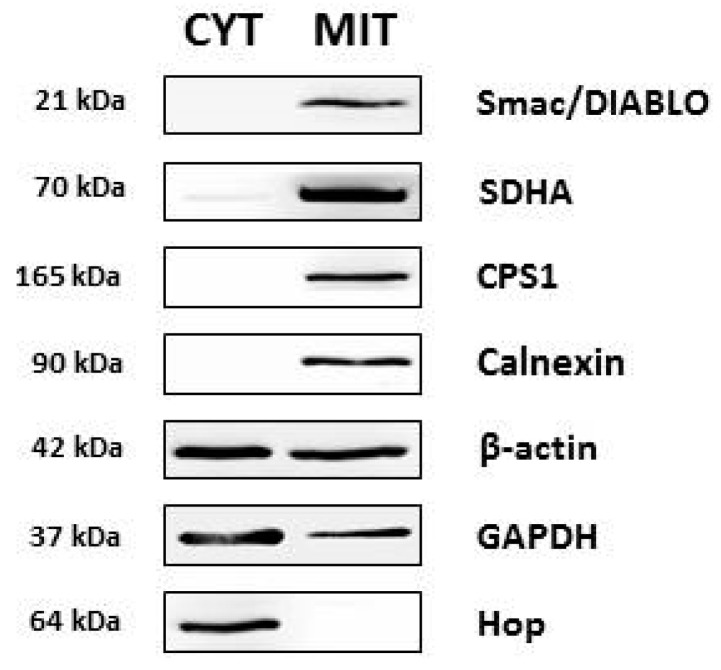
Comparison of the expression of selected proteins in cytosolic (CYT) and high-purity mitochondrial (MIT) fractions isolated from MCF7 cells using a QProteome Mitochondria Isolation Kit (see Section 4). The level of tested proteins was assessed using western blot analysis employing relevant antibodies (see Section 4). Smac/Diablo (Second mitochondria-derived activator of caspases/Direct IAP binding protein with low pI, mitochondrial intermembrane space), SDHA (succinate dehydrogenase complex flavoprotein subunit α, inner mitochondrial membrane), CPS1 (carbamoyl-phosphate synthetase 1, mitochondrial matrix), calnexin (endoplasmic reticulum), β-actin (representative cytoskoletal protein), GAPDH (glyceraldehyde 3-phosphate dehydrogenase, cytosol), Hop (Hsp70-Hsp90 organizing protein, cytosol).

At first, we tested the integrity of the mitochondrial fraction from MCF7 cells using western blot analysis with antibodies against markers of mitochondrial compartments. We observed a CPS1 (carbamoyl-phosphate synthetase 1) (mitochondrial matrix marker) and Smac/Diablo (second mitochondria-derived activator of caspase/direct inhibitor of apoptosis-binding protein with low pI) (mitochondrial intermembrane marker) signal in the mitochondrial fraction but not in the cytosolic fraction. SDHA (succinate dehydrogenase flavoprotein complex, subunit alpha) (inner mitochondrial membrane marker) was also detected in the mitochondrial fraction as well as weakly detected in the cytosolic fraction (Figure 1). These data indicate that the mitochondrial fraction contains mostly undamaged mitochondria.

To examine potential contamination of the mitochondrial fraction, we performed western blot analysis using antibodies against the endoplasmic reticulum, cytoskeleton, and cytosolic proteins. The mitochondrial fraction contained calnexin (an endoplasmic reticulum protein), β-actin (cytoskeletal protein), and GAPDH (glyceraldehyde-3-phosphate dehydrogenase) (Figure 1). Other cytoplasmic proteins, such as Hop (Hsp70-Hsp90 organizing protein) and Akt/PKB (protein kinase B) were virtually absent in the mitochondrial fraction. Taken together, the mitochondrial fraction was free of most cytosolic proteins. However, contamination with endoplasmic reticulum as well as cytoskeleton proteins was detected.

### 2.2. Two-Dimensional Electrophoresis of Mitochondrial Fraction

**Figure 2:**
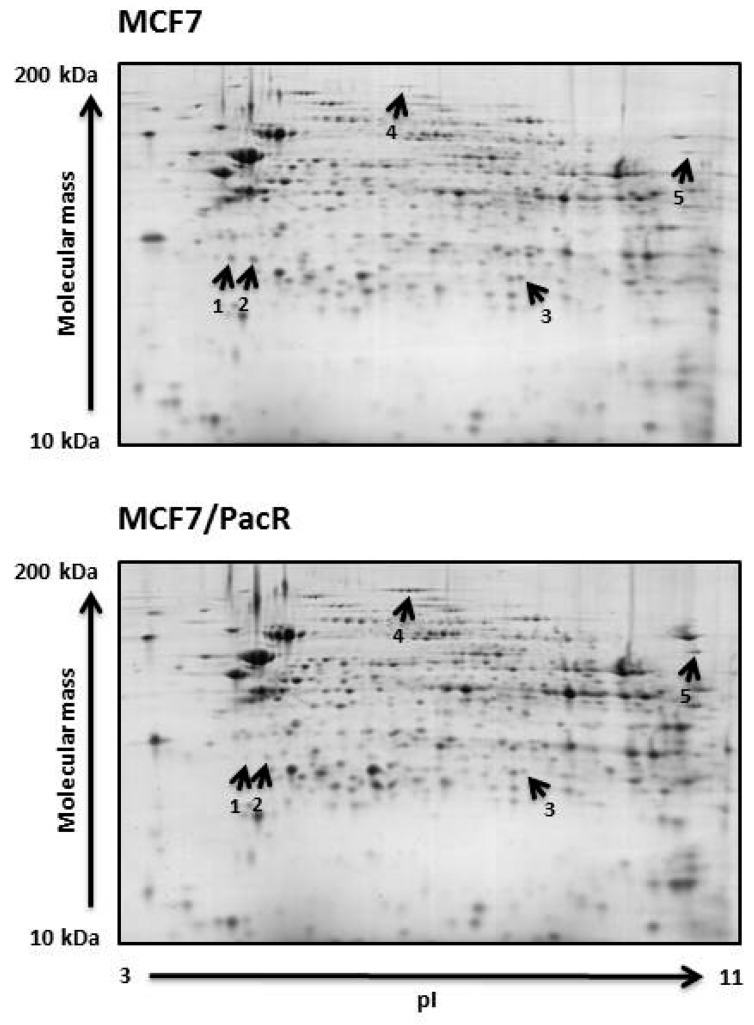
Differences between paclitaxel-sensitive MCF7 cells and paclitaxel-resistant MCF7/PacR cells concerning protein expression in high-purity mitochondrial fractions. Representative 2D gels of three independent pairs of gels (see Section 4) show five spots with differing expression (at least two-fold change). These five spots were identified as cathepsin D (spot 1 and spot 2), ABHD11 (abhydrolase domain-containing protein 11) (spot 3), CPS1 (carbamoyl-phosphate synthetase 1) (spot 4), and ATAD3A, ATAD3B (ATPase family AAA domain-containing protein 3A and 3B) (spot 5).

We used the “high-purity mitochondrial fraction” of paclitaxel-sensitive MCF7 and paclitaxel-resistant MCF7/PacR cells for conventional 2D-PAGE analysis (see Section 4). Coomassie brilliant blue-stained pairs of gels (3-11NL, 11 cm, 125 μg of proteins) from MCF7 (served as controls) and MCF7/PacR cells, were used for spot analyses. Three independent pairs of gels were used. In these gels, we were able to detect and match approximately 600 spots per gel (Figure 2) using ImageMaster 2D Platinum 6.0 software (see Section 4). It should be noted that the observed number of spots did not correspond to the number of detected proteins. This was mainly due to post-translational modifications that affected the isoelectric point as well as the molecular weights of the proteins. Thus, some proteins occurred as multiple spots in 2D-PAGE gel, which were typically seen as a string of spots.

### 2.3. Spot Analysis and Protein Identification

Spot volumes of 600 matched spots were statistically analyzed (Student´s *t*-test). We found five spots with altered (at least a two-fold change) spot volumes in MCF7/PacR cells compared to control MCF7 cells (Figure 2). These spots were excised and digested with trypsin protease. The resulting peptides were analyzed using MALDI-TOF mass spectrometry (for details see Section 4).

**Figure 3:**
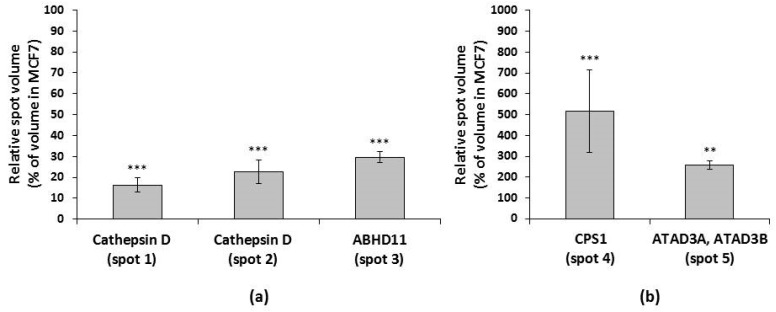
Expression levels of the identified proteins, (**a**) downregulated and (**b**) upregulated, in paclitaxel-resistant MCF7/PacR cells is presented as a percentage of the level in paclitaxel-sensitive MCF7 cells. Each column represents the mean value of the expression level ± SEM of the corresponding spots from three independent sets of gels. ** *p* < 0.01, *** *p* < 0.001 when compared with the level in MCF7 cells.

Spots with downregulated expression in MCF7/PacR cells were lysosomal cathepsin D (spot 1 and 2 with volumes decreased to 16% and 23% of control cells, respectively), mitochondrial abhydrolase domain-containing protein 11 (ABHD11) (spot 3 with a volume decreased to 30% of control cells). Spots with upregulated expression were mitochondrial carbamoyl-phosphate synthetase 1 (CPS1) (spot 4 with a volume increased to 518% of control cells), mitochondrial ATPase family AAA domain-containing protein 3A and 3B (ATAD3A, ATAD3B) (spot 5 with a volume increased to 257% of control cells) (Figure 2 and Figure 3, Table 1).

MALDI-TOF MS data indicated that the cathepsin D spots (spots 1 and 2, approximately 28 kDa) represent the heavy-chain of catalytically active full-form cathepsin D (45 kDa) based on their ascertained sequences and molecular weight [30] (Table 1, Figure 2). These spots differ in their isoelectric point (likely due to post-translational modification). For both spots, the experimental isoelectric point (approximately 4.7 and 5.0) is lower than theoretical (6.1).

The spot determined as ABHD11 (spot 3) also differed from its predicted (35 kDa) molecular weight (Table 1). We estimated its molecular weight to be approximately 27 kDa based on its position relative to the cathepsin D heavy chains spots (28 kDa) in 2D-PAGE gels. The experimental isoelectric point (approximately 7.2) of ABHD11 significantly differs from theoretical (9.5).

Spot 4 (a string of five spots of same molecular weight) (Table 1, Figure 2) were determined to be CPS1, which likely belongs to the post-translationally modified full-length protein (we estimated the molecular weight to be more than 150 kDa). The experimental isoelectric point is in agreement with theoretical value (6.3).

Mitochondrial proteins ATAD3A and ATAD3B (spot 5) are products of two genes (ATAD3A and ATAD3B) and their molecular weight (71 and 73 kDa, respectively) corresponded to their position in the 2D-PAGE gels. The experimental isoelectric point (approximately 10.4–10.7) differs from theoretical values (9.1 and 9.3).

### 2.4. Western Blot of Identified Proteins

**Figure 4:**
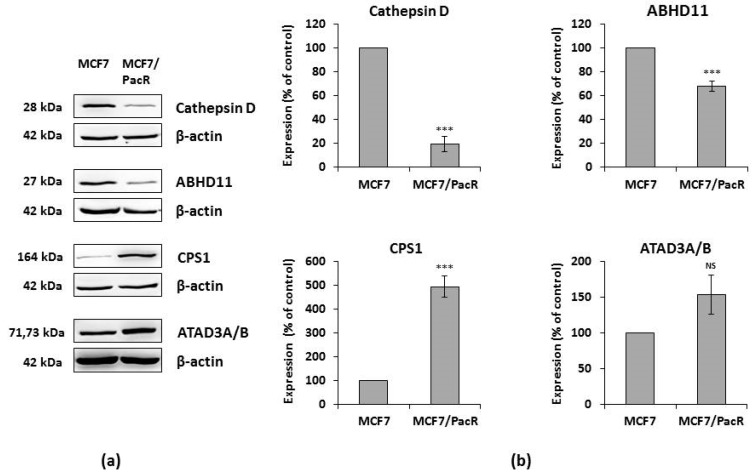
Confirmation of changes in protein expression detected by 2D analysis employing western blot analysis. (**a**) Western blot was performed with whole-cell lysates of paclitaxel-sensitive MCF7 cells and paclitaxel-resistant MCF7/PacR cells. Levels of tested proteins were assessed using western blot analysis employing relevant antibodies (see Section 4). β-actin served as a loading control. Representative results are from three independent experiments. (**b**) Densitometric analysis of western blots normalized to β-actin level. The expression of proteins in MCF7/PacR was compared to the expression in MCF cells (100%), and relative values were normalized to β-actin levels. Columns represent mean values of band volume ± SEM from three experimental values. *** *p* < 0.001 compared to the volume in MCF7 cells. NS = statistically non-significant difference.

We validated the changed expression of identified proteins using western blot and subsequent densitometry. We used whole-cell lysates of MCF7 cells and MCF7/PacR cells. Expression of cathepsin D (28 kDa band) decreased to 19% in MCF7/PacR cells (compared to control sensitive cells). This band should represent both spot 1 and spot 2 due to similar molecular weights. Expression of ABHD11 (27 kDa) decreased to 68% in MCF7/PacR cells, and expression of CPS1 (164 kDa) increased to 494% in MCF7/PacR cell. Expression of ATAD3A/B (71–73 kDa) increased to 154% in MCF7/PacR cells, however, the densitometric measurement was statistically insignificant in this case (Figure 4).

### 2.5. Distribution of CPS1 within Cells

**Figure 5:**
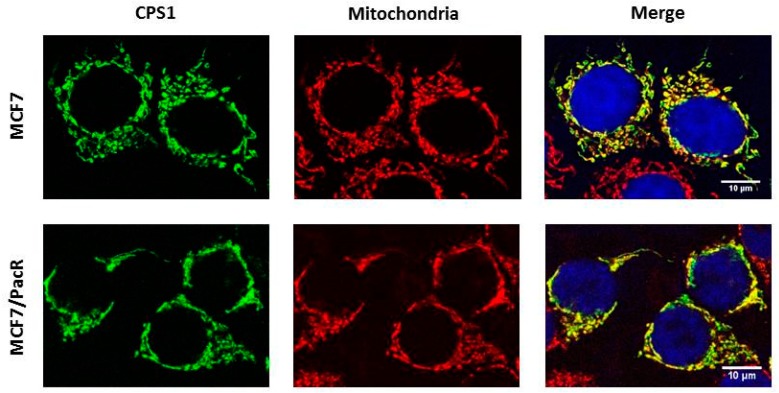
Cellular distribution of CPS1 (carbamoyl-phosphate synthetase 1) in paclitaxel-sensitive MCF7 cells and paclitaxel-resistant MCF7/PacR cells. The localization of CPS1 was detected using confocal microscopy (see Section 4). The localization of CPS1 (green), mitochondria (red), nuclei (blue) and the merge are shown. The data shown were obtained in one representative experiment of two independent experiments.

In order to assess the distribution of CPS1, which was the most upregulated protein in MCF7/PacR cells, we used confocal microscopy. Colocalization with the mitochondrial marker cytochrome c oxidase subunit IV (Cox IV) showed localization of CPS1 in the mitochondria of MCF7 cells as well as MCF7/PacR cells. It has been proposed [37] that CPS1 is also localized in the cell nucleus. However, we did not detect CPS1 in the nuclei of either MCF7 and MCF7/PacR cells (Figure 5). 

**Figure 6:**
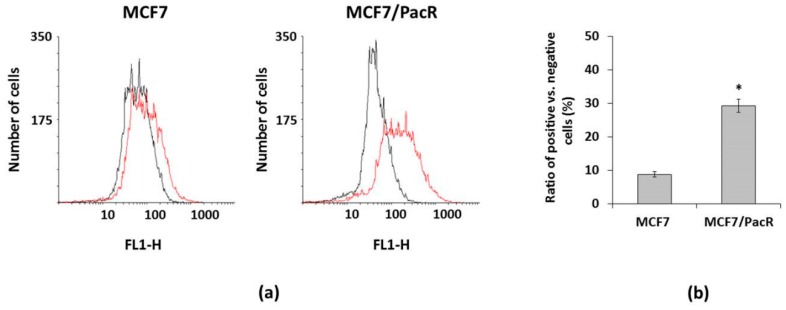
Expression of CPS1 (carbamoyl-phosphate synthetase 1) in paclitaxel-sensitive MCF7 cells and paclitaxel-resistant MCF7/PacR cells. The expression was assessed employing FACS (see Section 4). The data shown were obtained in one representative experiment from three independent experiments. (**a**) Histograms of MCF7 and MCF7/PacR cells, which were stained with a secondary antibody (black) or stained with a specific CPS1 antibody and then with the secondary antibody (red). (**b**) The number of CPS1 positive cells vs. negative cells (ratio) in MCF7 and MCF7/PacR cell population. Columns represent the mean value of the ratio ± SEM from two experimental values. * *p* < 0.05 compared to the ratio in paclitaxel-sensitive MCF7 cells.

By using flow cytometry, we detected increased levels of CPS1 in MCF7/PacR cells (Figure 6a). However, the observed differences were due to the different number of CPS1 positive cells in MCF7 and MCF7/PacR cell populations. In MCF7 cells, only 9% were CPS1 positive cells whereas the number of CPS1 positive cells increased significantly to 30% in MCF7/PacR cells (Figure 6b). Thus, most MCF7, as well as MCF7/PacR cells, did not express CPS1. Upregulated expression of CPS1 is rather caused by the increasing number of CPS1 positive MCF7/PacR cells and not due to the increase of CPS1 expression in each MCF7/PacR cell.

### 2.6. Effect of CPS1 Silencing on Resistance to Paclitaxel

**Figure 7:**
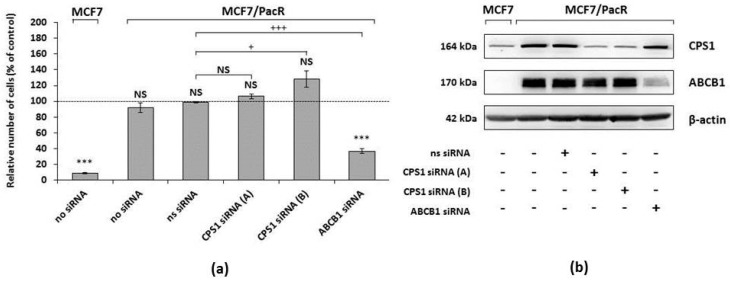
The effect of CPS1 (carbamoyl-phosphate synthetase 1) silencing and ABCB1 (ATP-binding cassette transporter B1) silencing on the growth and survival of paclitaxel-resistant MCF7/PacR cells in the paclitaxel-containing medium compared with the growth and survival of sensitive MCF7 cells in the paclitaxel-containing medium. (**a**) The cells were prepared and seeded as described in “Materials and Methods”. The relative number of living sensitive MCF7 cells (no siRNA), resistant MCF7/PacR cells (no siRNA), resistant cells treated with non-specific siRNA (ns siRNA), and resistant cells treated with two different (A and B) CPS1 specific siRNAs (CPS1 siRNA), as well as with an ABCB1 specific siRNA (ABCB1 siRNA), was determined after 96 h of incubation (the number of sensitive MCF7 or resistant MCF7/PacR cells grown in paclitaxel-free medium represents 100%, i.e., the control). Each column represents the mean ± SEM of three independent experiments. *** *p* < 0.001 compared to the control. NS = a statistically non-significant difference. ^+^ *p* < 0.05, ^+++^ *p* < 0.001 compared to the effect of ns siRNA. (**b**) The effect of ns siRNA, specific CPS1 siRNA (A) and CPS1 siRNA (B), as well as specific ABCB1 siRNA, on the expression of CPS1 and ABCB1 in sensitive MCF7 cells and resistant MCF7/PacR cells cultured in paclitaxel-containing medium, is also shown. Levels of tested proteins were assessed using western blot analysis and the relevant antibodies (see Section 4). β-actin served as a loading control. Representative results come from three independent experiments.

We further tested the effect of CPS1 silencing on the resistance of MCF7/PacR cells to paclitaxel. The effect was compared with the documented effect of ABCB1 silencing [27]. CPS1 and ABCB1 were knocked down in MCF7/PacR cells using Silencer® Select siRNAs (see “Materials and Methods”). Both used specific CPS1 siRNAs (A and B) efficiently (≥90%) silenced the expression of CPS1 in MCF7/PacR cells. ABCB1 knockdown was efficient to a similar extent. As a siRNA transfection control, we used MCF7/PacR cells treated with nonspecific siRNA (Figure 7b).

MCF7/PacR cells with silenced CPS1 (CPS1 siRNA A and B) or with silenced ABCB1 were cultivated in a paclitaxel-containing medium (i.e., a 300 nM, death-inducing, concentration of paclitaxel) or paclitaxel-free medium (control representing 100% of grown cells) for 96 hours. After 96 hours, the number of living cells was determined (see “Materials and Methods”). The number of non-treated MCF7 cells, incubated with 300 nM paclitaxel for 96 hours, decreased to 9%. The number of non-treated MCF7/PacR cells, incubated with 300 nM paclitaxel for 96 hours, slightly and non-significantly decreased to 92%. The number of MCF7/PacR cells treated with nonspecific siRNA in the paclitaxel-containing medium did not change. The numbers of MCF7/PacR cells treated with both (A and B) specific CPS1 siRNAs nonsignificantly increased (106% and 128%, respectively). The number of MCF7/PacR cells treated with specific ABCB1 siRNA significantly decreased to 37% (Figure 7a).

We compared the number of MCF7/PacR cells treated with nonspecific siRNA (siRNA transfection control) with the number of MCF7/PacR cells treated with specific CPS1 or ABCB1 siRNAs in order to test the effect of CPS1 knockdown on resistance to paclitaxel compared to the effect of ABCB1 knockdown. There was no statistically significant decrease in the number of MCF7/PacR cells treated with both specific CPS1 siRNAs (A and B). Furthermore, for CPS1 siRNA B there was a slight, but statistically significant increase. In comparison, MCF7/PacR cells with knocked down ABCB1, the number of grown cells significantly decreased (Figure 7a).

These data indicate that knockdown of CPS1 does not affect the resistance of MCF7/PacR cells to paclitaxel. In contrast to CPS1, the effect of ABCB1 siRNA silencing on the resistance of MCF7/PacR cells to paclitaxel was significant.

## 3. Discussion

In this paper, we studied potential differences in the expression of mitochondrial proteins between paclitaxel-sensitive MCF7 and paclitaxel-resistant MCF7/PacR breast cancer cells. For 2D-PAGE analysis, we used a mitochondrial fraction isolated using a commercial kit. The mitochondrial fraction was contaminated with endoplasmic reticulum and other cytosolic proteins, such as GAPDH, although the isolated mitochondria were nearly undamaged (Figure 1). Contamination with the endoplasmic reticulum is typical for mitochondria-enriched fractions due to the close, functional contact with mitochondria [38]. It has also been shown that GAPDH can be localized in various cellular compartments depending on its function [39]. Thus, the mitochondrial fractions also likely contained vesicles of non-mitochondrial origin and endoplasmic reticulum.

Therefore, it is not surprising that in addition to the mitochondrial proteins, we found lysosomal protein cathepsin D during the 2D-PAGE analysis (Figure 2, Table 1) likely as a result of lysosomal contamination of the mitochondrial fraction. Cathepsin D is a lysosomal aspartyl endoproteinase whose mature form consists of non-covalent interactions between light (14 kDa) and heavy (34 kDa) chains [40]. The heavy chain of cathepsin D (approximately 28 kDa in our study) was shown to be downregulated in paclitaxel-resistant MCF7/PacR cells (to 25% of the level in paclitaxel-sensitive MCF7 cells) [30]. In the present study, cathepsin D most likely represents a contamination product in the mitochondrial fraction. Unlike our previous study [30], we observed two similarly downregulated spots for the heavy chain of cathepsin D (Figure 2 and Figure 3, Table 1). The described spots differ only in their isoelectric point, likely reflecting their different post-translational modifications. Cathepsin D is known to be overexpressed in breast cancer cells, where it promotes growth and metastatic activity [41,42]. Signaling, via occupied intracellular estrogen receptors, stimulates cathepsin D transcription [43]. Tamoxifen was found to decrease cathepsin D expression [44]. Apart from its enzymatic role inside the lysosomes and in the tumor cell microenvironment (the secreted form of cathepsin D), it is also well known to be involved in the regulation of cell death [45,46,47]. Upon diverse death signals, cathepsin D translocates from lysosomes into the cytosol, where it executes its pro-apoptotic activities by cleaving specific substrates. The cleavage of these substrates contributes to the activation of the pro-apoptotic Bax protein [48]. Moreover, cathepsin D can directly cleave anti-apoptotic Bcl2 protein [49].

ABHD11 (abhydrolase domain-containing protein 11) also known as Williams–Beuren syndrome chromosomal region 21 protein (WBSCR21), is a member of the α/β-hydrolase domain family. Members of this large family are encoded by 19 human genes [50]. ABHD11 is localized in the locus that is deleted in Williams–Beuren syndrome, which is a multisystemic genetic disease [51]. It is unclear, whether ABHD11 deletion contributes to any of the symptoms associated with this syndrome. ABHD11 is a mitochondrial matrix hydrolase that processes an unknown lipid substrate [52]. Its predicted molecular mass (35 kDa) is in contrast with the observed position of the spot in our study (approximately 27 kDa) (Figure 2). This difference could be the result of the processing of ABHD11 N-terminal targeting sequence, at leucine 59, by mitochondrial processing peptidase during its transport into the mitochondria (based on MitoFates software, [53]). ABHD11 processing explains the discrepancy between its theoretical and experimental isoelectric point (Figure 2, Table 1). Processed N-terminal sequence is rich in basic amino acids (theoretical pI is 12.5). Increased enzymatic activity of ABHD11 was found in non-small lung cancer [54]. The ABHD11 locus also encodes for long non-coding RNA, termed as ABHD11-AS1 (antisense 1), whose expression is increased in gastric [55], colorectal [56], pancreatic [57] and endometrial [58] cancer. In MCF7/PacR cells, ABHD11 is downregulated (to 68% of the level in control MCF7 cells) (Figure 4). Heat-shock protein 27, which is encoded by a neighboring gene, was also downregulated (50% of the level in control MCF7 cells) [30].

CPS1 (carbamoyl-phosphate synthetase 1) was the most upregulated mitochondrial protein found in MCF7/PacR cells (490% of the level in MCF7 cells) (Figure 4 and Figure 5). As a mitochondrial matrix enzyme (EC 6.3.4.16), CPS1 catalyzes the first rate-limiting step of the urea cycle [59]. In this reaction, CPS1 generates carbamoyl phosphate from ammonia and bicarbonate. Recently, the CPS1 structure was elucidated in the presence of its activator, N-acetyl-L-glutamate (NAG) [59]. Unlike its prominent role in the urea cycle, there are currently no data about the participation of CPS1 in the acquired resistance to any chemotherapeutics. Remarkably, it was shown that transcription of CPS1 is negatively regulated by liver kinase B1 (also known as serine/threonine kinase 11) in lung adenocarcinoma cell lines [60,61]. In MCF7 cells, this kinase is regulated by estrogen [62]. The excess of mitochondrial carbamoyl phosphate can supply, by an unknown mechanism, the cytosolic pool that is utilized in pyrimidine synthesis [61], as well as promote cell proliferation. CPS1 could play a different role depending on the type of cancer cells. One study showed that overexpression of CPS1 associates with poor chemoradiotherapy response in rectal cancer [63]. However, in cancers of non-small intestinal origin, CPS1 expression is totally lost [64].

CPS1 gene is localized on chromosome 2 (Table 2) in the vicinity of the gene coding for a microtubule-associated protein (MAP2) which is a regulator of microtubule flexibility [65] and is known to be associated with resistance to paclitaxel [66]. Notably, we found that changes in CPS1 expression in MCF7/PacR cells (found by 2D-PAGE and western blot) (Figure 2, Figure 3 and Figure 4, Table 1) are likely caused by an increased number of cells expressing CPS1 (Figure 6). However, for most MCF7, as well as MCF7/PacR cells, the expression of CPS1 was at a low level. Silencing of CPS1 expression in resistant MCF7/PacR cells resulted in a non-significant change in the number of MCF7/PacR cells cultivated in paclitaxel-containing medium (Figure 7). However, the high expression of ABCB1 transporter in MCF7/PacR cells could have masked the effect of specific CPS1 siRNA. Therefore, we also performed a simultaneous knock-down of ABCB1 and CPS1 in MCF7/PacR cells. However, the data were confusing, likely due to the strong off-target effects of the siRNA combination. Thus, it remains a question of whether CPS1 plays any role in acquired resistance to paclitaxel.

ATAD3A and ATAD3B (ATPase family AAA-domain containing protein 3A and 3B) are proteins associated with the inner mitochondrial membrane, which are coded by nuclear genes [67]. In primates, the ancestral gene ATAD3A (coding for 71 kDa protein), was tandemly duplicated and mutated. It resulted in the novel ATAD3B (coding for 73 kDa protein) and ATAD3C gene (coding for 46 kDa protein) [68]. In MCF7/PacR cells, we detected both the ATAD3A and ATAD3B proteins in a single spot (with upregulation to 250% of the level in control MCF7 cells) (see Figure 2 and Figure 3, Table 1). However, we were not able to confirm the overexpression using western blot of whole cell lysates (results were statistically insignificant) (see Figure 4). ATAD3A forms hexameric ring structures, exposing its C-terminal ATPase domain into the mitochondrial matrix [69]. ATAD3 is crucial for maintaining mitochondrial dynamics [70], the mitochondrial nucleoid [71,72], the mitochondria-endoplasmic reticulum connection [73], cholesterol metabolism [74], cristae structure [74], and chemoresistance to doxorubicin [75]. ATAD3A expression correlates with the response to chemoradiotherapy in primary glioblastoma multiforme [76]. Higher expression of ATAD3A is associated with cisplatin resistance and PSA level in prostate cancer [77]. In breast and colon cancer, ATAD3A forms and stabilizes WASF3 in a complex with endoplasmic protein GRP78 [73]. WASF3 dysregulates expression of KISS3, a regulator of NFκB signaling pathway and thus promotes cell proliferation and metastatic activity [73]. It was shown that ATAD3B is expressed in pluripotent embryonic stem cells and in cancer cells where it negatively regulates ATAD3A function [78].

Multiple mechanisms for the regulation of ABCB1 expression in cancer cell lines have been discussed [79]. Interestingly, we showed that several genes (HSP27, ABHD11, ABCB4, and TRIP6) (Table 2), located on the long arm of chromosome 7 close to the ABCB1 and ABCB4 genes, have altered expression in MCF7/PacR cells. Curiously, the genes localized between centromere and ABCB1 (e.g., HSP27 and ABHD11) are both underexpressed and the gene for TRIP6, localized between the telomere and ABCB1, is overexpressed in MCF7/PacR. Changes in the expression of the mentioned genes could reflect genetic changes (like chromosome rearrangement) occurring in the long arm of chromosome 7.

## 4. Materials and Methods

### 4.1. Materials

All reagents were purchased from Sigma-Aldrich (St. Louis, MO, USA) unless otherwise specified. The following primary and secondary antibodies were used to detect protein expression: anti-CPS1 (B1) (sc376190, 1:500) from Santa-Cruz Biotechnology (Santa Cruz, CA, USA), anti-actin (clone AC-40, dilution 1:1000), anti-CPS1 [EPR7493-3] (ab129076, 1:1000), anti-GAPDH (ab9485, 1:1000) from Abcam (Cambridge, UK), anti-SDHA (D6J9M) XP® (#11998, 1:1000), anti-Smac/Diablo (79-1-83) (#2954, 1:1000), anti-COX IV (#11967S, 1:200), anti-Hop (D10E2) (#5670, 1:1000), anti-Calnexin (C5C9) (#2679, 1:1000) from Cell Signaling Technology (Danvers, MA, USA). Secondary antibodies HRP-linked goat anti-rabbit (sc-2004, 1:5000) or goat anti-mouse (sc-2005, 1:5000) were from Santa-Cruz Biotechnology (Santa Cruz, CA, USA). Secondary goat anti-rabbit IgG H&L Alexa Fluor®488 (ab150077) was from Abcam (Cambridge, UK), and goat anti-mouse IgG H&L F(ab)_2_ Alexa Fluor®594 (#8890S) was from Cell Signaling Technology (Danvers, MA, USA).

### 4.2. Cell Culture

Human breast cancer cell line MCF7 was purchased from ATCC (Manassas, VA, USA). The establishment of the paclitaxel-resistant subline MCF7/PacR was described previously [14]. The MCF7 breast cancer cell line was cultivated in RPMI medium supplemented with 10% fetal bovine serum, whereas the MCF7/PacR subline was cultivated in RPMI medium containing 10% FBS and 300 nM paclitaxel. The cells were cultivated at 37 °C in a humidified atmosphere with 5% CO_2_.

### 4.3. Preparation of Mitochondria-Enriched Fraction

For isolation of the mitochondria-enriched fraction, cells were seeded at a density of 2.0 × 10^6^ cells into six Petri dishes (Ø 10 cm, i.e., 1.2 × 10^7^ cells/experiment). After 24 h, media were replaced for the fresh ones. After 72 h, the cells were harvested with the trypsin-EDTA solution into 10 mL of ice-cold PBS and centrifuged (500 *g*, 10 min, 4 °C). Mitochondria were enriched using a QProteome® mitochondria isolation kit (Qiagen, Hilden, Germany) according to the manufacturer´s protocol with the following modifications. Briefly, cell pellets (approximately 6.0 × 10^7^ cells) were washed with 3 ml of 0.9% NaCl and resuspended in 3 ml of lysis buffer (with protease inhibitors). The resuspended cells were then incubated on an end-on-end shaker for 10 min at 4 °C and centrifuged (1000 *g* for 10 min, 4 °C). The supernatant (cytosolic fraction) was carefully removed for further analysis. Cell pellets were resuspended in 3 ml of disruption buffer (with protease inhibitors), and organelles were released by passing the suspension ten times through a 26 G needle. Samples were centrifuged (1000 *g*, 10 min, 4 °C) and the supernatant was transferred into new tubes. Cell pellets were resuspended in 1 mL of disruption buffer (with protease inhibitors), and the lysis was repeated by passing the suspension ten times through a 26 G needle. Samples were centrifuged (1000 *g*, 10 min, 4 °C), cell pellets (unbroken cells and nuclei) were kept for further analysis. Supernatants were mixed and centrifuged (6000 *g*, 10 min, 4 °C). The resulting supernatant (microsomal fraction) was kept for further analysis. Cell pellets (crude mitochondria fractions) were dissolved in 750 μL of mitochondria purification buffer and purified using a density gradient according to the manufacturer´s protocol. Finally, the pellets were resuspended in RIPA buffer (for western blot analysis) or protein extraction buffer V (for 2D-PAGE).

### 4.4. Two-Dimensional Electrophoresis

Pellets containing mitochondria-enriched fractions were resuspended in protein extraction buffer V (PEB V) with protease and phosphatase inhibitors and nuclease mix (GE Healthcare, Uppsala, Sweden). Protein concentrations were determined using a 2-D quant kit (GE Healthcare, Uppsala, Sweden). Due to the low amounts of isolated proteins in each sample, lysates were not cleaned using the precipitation method (i.e., 2-D Clean-Up Kit). Each sample (120 μg of proteins) was mixed with 4 μL bromophenol blue solution 0.1%, 2 μL IPG buffer 3-11NL (ampholytes), and 4 μL Destreak reagent (GE Healthcare, Uppsala, Sweden). Samples were passively rehydrated onto 11 cm 3-11NL IPG strips (GE Healthcare, Uppsala, Sweden) at 20 °C for at least 24 h. Isoelectric focusing was performed using an Ettan IPGPhor II unit with the following protocol: gradient 150 V for 1 h → 150 V for 1 h → gradient 300 V for 0.5 h → 300 V for 2 h → gradient 1500 V for 2.5 h → 1500 V for 1 h → gradient 5000 V for 5 h → 5000 V for 9 h. The total voltage-hour was 65 KV·h.

After IEF, the strips were equilibrated in equilibration (EQ) buffer (6 M urea, 30% glycerol, 2% SDS, 50mM Tris-HCl pH 8.8) containing 2% DTT (EQ-DTT) for 20 min at room temperature and then equilibrated in EQ buffer containing 2.5% iodoacetamide (EQ-IAA) using the same conditions. Polyacrylamide gels (12% pH 8.8 resolving, and 4% pH 8.8 stacking gels) were hand cast in criterion empty cassettes (13.3 × 8.7cm) (Biorad, Hercules, CA, USA). The equilibrated strips were placed on top of the stacking gels and covered with agarose sealing solution (0.5% agarose, Laemmli running buffer, bromophenol blue). The electrophoresis was run at a constant voltage of 60 V.

Gels were rinsed three times with distilled water for 5 min and stained overnight in 100 mL colloidal Coomassie Brilliant Blue G-250 (distilled water, 10% ethanol, 2% orthophosphoric acid, 0.02% Coomassie Brilliant Blue G-250). Gels were rinsed three times with distilled water and destained in destaining solution (10% ethanol, 2% orthophosphoric acid, distilled water) for 20 min. Gels were stored in 1% acetic acid at 4°C.

### 4.5. Analysis of Two-Dimensional Electrophoresis Gels

Gels were scanned using a UMAX PowerLook 1120 scanner and Labscan software (GE Healthcare, Uppsala, Sweden) at 600 dpi. Images were analyzed using Image Master^TM^ 2D Platinum 6.0 software (GE Healthcare, Uppsala, Sweden). Spots were manually detected and analyzed using three independent sets of gels (sensitive MCF7 versus paclitaxel-resistant MCF7/PacR). The statistical significance of the change in expression of matched spots was determined using the Student´s *t*-test. Spots with at least a two-fold change in the signal were selected for MALDI-TOF mass spectrometry (MS) analysis.

### 4.6. Mass Spectrometry

Mass spectrometry was performed as previously described [30]. Cut spots were destained with 50 mM 4-ethylmorpholine acetate (pH 8.1) in 50% acetonitrile (MeCN). After complete destaining, gels were washed with water, reduced in size via dehydration in MeCN and reconstituted again in water. The supernatant was removed, and gels were partly dried in a SpeedVac concentrator. Gel pieces were then incubated overnight at 37 °C in a cleavage buffer containing 25 mM 4-ethylmorpholine acetate, 5% MeCN, and trypsin (100 ng, Promega, Madison, WI, USA). The resulting peptides were extracted with 40% MeCN/0.1% trifluoroacetic acid (TFA).

An aqueous 50% MeCN/0.1% TFA solution of α-cyano-4-hydroxycinnamic acid (5 mg/mL, Sigma-Aldrich, St. Louis, MO, USA) was used as a MALDI matrix. The peptide mixture (1 μL) was deposited on a MALDI plate, allowed to air-dry at room temperature and overlaid with 0.4 μL of the matrix. Mass spectra were measured using an Ultraflex III MALDI-TOF (Bruker Daltonics, Bremen, Germany), mass range of 700–4000 Da, calibrated internally using monoisotopic [M+H]^+^ ions of trypsin auto-proteolytic fragments (842.5 and 2211.1 Da). The peak lists, created using the flexAnalysis 3.3 program, were searched using an in-house MASCOT search engine against the SwissProt 2013_12 database subset of human proteins with the following search settings: peptide tolerance of 30 ppm, missed cleavage site value set to one, variable carbamidomethylation of cysteine, oxidation of methionine and protein N-term acetylation. Proteins with MOWSE scores over the threshold of 56 (calculated for the settings used) were considered as identified. The identities of protein candidates were determined using MS/MS analysis.

### 4.7. Whole-Cell Lysate Preparation

Whole-cell lysates were prepared from cells growing on Petri dishes (Ø 6 cm). The cells (approximately 3.0 × 10^6^) were harvested with trypsin-EDTA solution into 10 mL of ice-cold phosphate-buffered saline (PBS) and centrifuged (500 *g*, 10 min, 4 °C). Cell pellets were washed twice with ice-cold PBS and centrifuged (500 *g*, 10 min, 4 °C). The resulting pellets were resuspended in RIPA™ buffer (Merck Millipore, Burlington, MA, USA) using the cOmpleteTM Mini protease inhibitor cocktail mix (Roche, Rotkreuz, Switzerland). After incubation for 20 min on ice, the lysate was centrifuged (16,000 *g*, 20 min, 4 °C) and the supernatant (whole-cell lysate) was transferred into a new tube. Protein concentrations were determined using a Pierce BCA Protein Assay Kit (Thermo Fisher Scientific, Waltham, MA, USA).

### 4.8. Western Blot Analysis

Immunoblotting was performed as previously described [28,80,81]. Whole-cell lysates samples (20 μg) or cell fractions (12 μg) were mixed with sample buffer (0.125M Tris/HCl pH6.8, 10% glycerol, 4% SDS, 0.25M DTT) and heated for 5 min at 95 °C. Samples were separated in 12% hand casted polyacrylamide gels using protein electrophoresis (Bio-Rad, Hercules, CA, USA). Separated proteins were blotted onto 0.2 μm nitrocellulose membranes PROTRAN BA 83 (Whatman-Schleicher and Schuell, Maidstone, UK) at 0.25 A for 3 h using a MiniProtean II blotting apparatus (Bio-Rad, Hercules, CA, USA). The membrane was blocked with 5% BSA in TBS for 30min and incubated with the primary antibody at 4 °C overnight. Membranes were washed three times with TBS containing 0.1% Tween-20 (TBS-T). Then the membranes were incubated for 2 h with the corresponding horseradish peroxidase-conjugated secondary antibody. The membrane was again washed three times with TBS-T. The chemiluminescence signal was detected using SuperSignal^TM^ West Pico PLUS Chemiluminescent Substrate (Thermo Fisher Scientific, Waltham, MA, USA) and a CCD camera GEL Logic 4000 Pro (Carestream Health, New Haven, CT, USA).

### 4.9. Confocal Microscopy

Cells were seeded at a density of 5–6 × 10^4^ cells onto coverslips. After an attachment period, the cells were fixed with 4% paraformaldehyde for 20 min, washed three times with PBS (10 min), and permeabilized with 0.3% Triton X-100 for 15–20 min. After three washing steps, the cells were blocked for 60 min with 1% BSA and stained with the primary antibodies diluted 1:200 in 1% BSA anti-CPS1 [EPR7493-3] (Abcam, Cambridge, UK) and anti-COX IV (#11967S) (Cell Signaling Technology, Danvers, MA, USA) at 4 °C overnight. The cells were then washed three times with PBS and incubated with secondary antibodies goat anti-rabbit IgG H&L Alexa Fluor®488 (ab150077) and goat anti-mouse IgG H&L F(ab)_2_ Alexa Fluor®594 (#8890S) (Abcam, Cambridge, UK) for 2 h in the dark, at room temperature. Finally, cells were washed again with PBS. Next, cells were transferred onto a droplet of Vectashield® Mounting Medium with DAPI (Vector Laboratories, Burlingame, CA, USA) and sealed. Samples were analyzed using a Leica TCS SP5 confocal microscope (Bannockburn, IL, USA).

### 4.10. FACS Analysis

The cells (approximately 2.5 × 10^6^ cells per sample) were seeded into Petri dishes. After an attachment period, cells were harvested into PBS and sedimented by low-speed centrifugation (500 *g*, 10 min, 4 °C). Next, cells were fixed by 3–4% formaldehyde at 37 °C for 10 min, centrifuged, washed with PBS, and permeabilized using 90% methanol on ice for 30 min. Subsequently, cells were washed with PBS. Nonspecific reactions were prevented by incubation in 3% BSA for 60 min. Cells were incubated with primary antibody anti-CPS1 [EPR7493-3] (Abcam, Cambridge, UK) diluted 1:80 in 3% BSA for 60 min. Then cells were washed with PBS and incubated 60 min with Goat anti-rabbit IgG H&L Alexa Fluor®488 (ab150077) (Abcam, Cambridge, UK) diluted 1:200 in 3% BSA. Finally, cells were washed with PBS, and the intensity of cell fluorescence was measured using a FACS Calibur cytometer (Becton Dickinson, San Jose, CA, USA). 

### 4.11. siRNA Silencing and Its Effect on Resistance to Paclitaxel

CPS1 expression was knockdown using Silencer® Select siRNA ID s3462 (“siRNA CPS1 A“, transcription variant 1, 3) and ID s528702 (“siRNA CPS1 B“ transcription variant 1, 2, 3). ABCB1 was knock-downed using Silencer® Select siRNA ID s10419. Nonspecific Silencer® Select siRNA 4390844 was used as a negative control. All siRNAs were purchased from Life Technologies (Carlsbad, CA, USA). Cells were transiently transfected with INTERFERin reagent (PolyPlus-Transfection, Illkirch, France) in Opti-MEM® Reduced Serum Medium (Life Technologies, Carlsbad, CA, USA) according to manufacturer instructions.

The cells were seeded at a density of 2.1 × 10^5^ cells per Petri dish (Ø 6 cm) in antibiotic-free medium. After 24 h both paclitaxel-sensitive (MCF7) and paclitaxel-resistant (MCF7/PacR) cells were transfected in paclitaxel-free medium. In the transfection mixture, CPS1, ABCB1, or non-specific siRNAs were diluted in Opti-MEM® Reduced Serum Medium to a final concentration of 5 nM of siRNA (for double siRNA transfection the final concentration was 10 nM siRNA in total, i.e., 5 nM each) in the culture medium together with the INTERFERin transfection reagent at a 1:250 dilution. After 72 h of incubation with siRNA, cells were harvested and seeded in the fresh media at a concentration of 2.0 × 10^5^ cells/ml into culture plates. After 24 h, allowing cells to attach, the media was replaced by media containing paclitaxel. After 96 h of treatment, the number of surviving cells was determined using a hemocytometer after trypan blue staining, and the persistence of CPS1 and ABCB1 silencing throughout the experiment was analyzed using western blot (see above).

### 4.12. Statistical Analysis

The statistical significance of differences was determined using the Student’s *t*-test. *p* < 0.05, *p*  < 0.01, and *p* < 0.001 were considered statistically significant at the 5%, 1%, and 0.1% levels, respectively. NS means statistically insignificant.

## 5. Conclusions

In this study, we detected three mitochondrial proteins (CPS1, ATAD3A/ATAD3B, and ABHD11) and one lysosomal protein (cathepsin D) with different expressions in paclitaxel-resistant MCF7/PacR breast cancer cells compared to paclitaxel-sensitive MCF7 breast cancer cells. None of these mitochondrial proteins have previously been associated with acquired resistance of breast cancer cells to paclitaxel.

In the case of mitochondrial ATAD3A and ATAD3B proteins, we detected increased expression in the spot containing peptides of both proteins in MCF7/PacR cells. However, this result was not confirmed with western blot and whole-cell lysates. Mitochondrial lipase ABHD11 was downregulated in MCF7/PacR cells. Owing to its position in human genome (i.e. on the long arm of chromosome 7, close to ABCB1, ABCB4, and HSP27), the change in ABHD11 expression likely reflect genetic changes (e.g., chromosome rearrangement) during selection of paclitaxel-resistant cells having a high expression of the ABCB1 transporter rather than its direct involvement in acquired resistance to paclitaxel. Downregulation of cathepsin D expression in MCF7/PacR cells is in accordance with its described role as an effector molecule in lysosomal cell death.

For CPS1, which was the most upregulated protein in MCF7/PacR cells, we observed the same mitochondrial localization, using confocal microscopy, of CPS1 in both sensitive and resistant cells. The role of CPS1 in acquired resistance to paclitaxel was not demonstrated during the CPS1 knockdown experiment, which used specific siRNA.

## Figures and Tables

**Table 1:** Protein identification of five spots with differing expression using MALDI-TOF MS. Table includes spot number, protein name, UniProtKB database number (DTB No.), number of peptides matched to the identified protein, sequence coverage (SC), peptide sequences confirmed by MS/MS, theoretical (Th.)/experimental (Exp.) values of protein molecular weight (MW) and pI.

Spot No.	Protein Name	DTB No.	No. of Peptides	SC [%]	MS/MS Confirmation	MW [kDa] Th./Exp.	pI Th./Exp.
1	Cathepsin D	P07339	12	33	FDGILGMAYPR	45/28	6.1/4.7
					YYTVFDRDNNR		
					LVDQNIFSFYLSR		
2	Cathepsin D	P07339	15	38	FDGILGMAYPR	45/28	6.1/5.0
					YYTVFDRDNNR		
					LVDQNIFSFYLSR		
					ISVNNVLPVFDNLMQQK		
3	Abhydrolase domain-containing protein 11, ABHD11	Q8NFV4	21	68	AINIADELPR	35/27	9.5/7.2
					GGAEPRPLPLSYR		
					TAMLLALQRPELVER		
					VNLDALTQHLDKILAFPQR		
4	Carbamoyl-phosphate synthase 1 [ammonia], mitochondrial, CPS1	P31327	14	10	FVHDNYVIR	164/164	6.3/6.0–6.2
					GILIGIQQSFRPR		
					SAYALGGLGSGICPNR		
5	ATPase family AAA domain-containing protein 3A, ATAD3A	Q9NVI7	29	38	TAGTLFGEGFR	71/71	9.1/10.4–10.7
					LDSVIEFSIPDSLLIR		
					LQAYHTQTTPLIEYYR		
					QRYEDQLKQQQLLNEENLR		
	ATPase family AAA domain-containing protein 3B, ATAD3B	Q5T9A4	17	29	LKEYEAAVEQLKSEQIR	73/73	9.3/10.4–10.7

**Table 2:** All identified protein expression change in paclitaxel-resistant MCF7/PacR cells compared to paclitaxel-sensitive MCF7 cells. The table includes protein name, protein localization within the cell, known or predicted function in the cell, localization of the gene within the human genome, expression level in MCF7/PacR cells (compared to the level in MCF7 cells) determined by western blot, and corresponding references.

Protein Name/Abbreviation	Protein Localization	Function	Gene Localizaton	Expression in MCF7/PacR Cells
ATP binding cassette subfamily B member 1, **ABCB1**	Plasma membrane	Drug efflux [14]	7q21.12	Overexpressed, concrete level not determined [14]
ATP binding cassette subfamily B member 4, ABCB4	Plasma membrane	Drug efflux [14]	7q21.12	Overexpressed, concrete level not determined [14]
ATP binding cassette subfamily C member 2, **ABCC2**	Plasma membrane	Drug efflux [14]	10q24.2	Overexpressed, concrete level not determined [14]
ATP binding cassette subfamily C member 3, **ABCC3**	Plasma membrane	Drug efflux [14]	17q21.33	Overexpressed, concrete level not determined [14]
Abhydrolase domain-containing protein 11, **ABHD11**	Mitochondria	Putative lipid hydrolase [52]	7q11.23	68% [this paper]
ATPase family AAA domain-containing protein 3A and 3B, **ATAD3A**, **ATAD3B**	Mitochondria	Multiple (mitochondrial network, cristae structure, nucleoid binding) [69,71,72,73,74,75]	1p36.33	154% [this paper]
Carbamoyl-phosphate synthetase 1, **CPS1**	Mitochondria	Enzyme (urea cycle) [59,61]	2q34	494% [this paper]
Cathepsin D, **CTSD**	Lysosomes	Protein degradation, cell death [40,48,49]	11p15.5	28% [30], 19% [this paper]
Heat shock protein family B (small) member 1, **HSP27**	Cytosol	Signaling [30]	7q11.23	47% [30]
Thyroid hormone receptor interactor 6, **TRIP6**	Cytosol	Antiapoptotic signaling [30]	7q22.1	650% [30]

## Abbreviations

2D-PAGE	Two-dimensional polyacrylamide gel electrophoresis
ABCB1	ATP-binding cassette transporter member B1
ABCB4	ATP-binding cassette transporter member B4
ABCC2	ATP-binding cassette transporter member C2
ABCC3	ATP-binding cassette transporter member C3
ABHD11	Alpha/beta hydrolase domain-containing protein 11
ATAD3A	ATPase family AAA domain-containing protein 3A
ATAD3B	ATPase family AAA domain-containing protein 3B
CPS1	Carbamoyl phosphate synthetase 1
COX IV	Cytochrome c oxidase subunit IV
Diablo	Direct inhibitor of apoptosis-binding protein with low pI
GAPDH	Glyceraldehyde-3-phosphate dehydrogenase
HER2	Human epidermal growth factor receptor 2
Hop	Hsp70-Hsp90 organizing protein
HSP27	Heat-shock protein 27
MALDI-TOF	Matrix-assisted laser desorption/ionization-time-of-flight
MAP2	Microtubule-associated protein 2
MCF7	Michigan Cancer Foundation 7
SDHA	Succinate dehydrogenase complex flavoprotein, subunit alpha
STK11	Serine/threonine kinase 11
Smac	Second mitochondria-derived activator of caspase
WBSCR21	Williams–Beuren syndrome chromosomal region 21 protein
